# Integrating the Meaning of Person Names into Discourse Context: An Event-Related Potential Study

**DOI:** 10.1371/journal.pone.0083206

**Published:** 2013-12-12

**Authors:** Lin Wang, Yufang Yang

**Affiliations:** Key Laboratory of Behavioral Science, Institute of Psychology, Chinese Academy of Sciences, Beijing, China; Lancaster University, United Kingdom

## Abstract

The meaning of person names is determined by their associated information. This study used event related potentials to investigate the time course of integrating the newly constructed meaning of person names into discourse context. The meaning of person names was built by two-sentence descriptions of the names. Then we manipulated the congruence of person names relative to discourse context in a way that the meaning of person names either matched or did not match the previous context. ERPs elicited by the names were compared between the congruent and the incongruent conditions. We found that the incongruent names elicited a larger N400 as well as a larger P600 compared to the congruent names. The results suggest that the meaning of unknown names can be effectively constructed from short linguistic descriptions and that the established meaning can be rapidly retrieved and integrated into contexts.

## Introduction

Person names are used to represent individuals. They are associated with faces, facts (e.g., ‘*John has a daughter.*’) or events (e.g., ‘*John celebrated his daughter’s birthday yesterday.*’) in the form of semantic or episodic memory. It has been proposed that the associated information constitutes the meaning of person names ([1], [2], [3]; but see [4]).

Name processing was proposed to involve several stages [5]: an initial word form analysis (i.e., word recognition), name recognition if the name is familiar, person identification (i.e., to link the name to the person), and finally, activation of associated information (e.g., the occupation of the name bearer). Therefore, the access of a names’ meaning takes place after word recognition, which is followed by name recognition and person identification. In contrast, the meaning of other categories of words (such as nouns, verbs and adjectives) can be accessed directly after word recognition, with no extra association process necessary [6]. That is, the access of a name's meaning (i.e., the activation of associated information) takes place after all three processes (word recognition, name recognition, and person identification) have finished, while only one process (word recognition) has to be completed for the access of word meaning. In this regard, the retrieval of known names should be slower than that of the other categories of words. Indeed, longer reaction times were reported for names than for nouns in a phonological decision task [7] in which participants were asked to silently retrieve the defined words (the frequency was matched) based on short, written, unequivocal definitions of names and nouns and thereafter to decide whether a specific syllable was part of the defined words. Moreover, our previous event-related potential (ERP) study found that the retrieval of the emotional meaning of known names (e.g., *Hitler* is negative while *Churchill* is positive) occurred around 640 ms, further indicating that the retrieval of the meaning of known names occurs rather late [8].

However, existing studies on person names have mainly focused on famous or familiar names. Unknown names, just like novel words, are meaningless to readers or listeners. A logical question then is how people can acquire the meaning of unknown names. It has been shown that people can derive the meaning of novel words (e.g., *lankey*) after several exposures of the novel words in a semantically constraining context (e.g., ‘*After a meal you should brush your lankey.*’; [9]), and that the meaning of the newly acquired word was deployed rapidly in sentence processing [10]. The successful mapping between novel words (e.g., *lankey*) and known concepts (e.g., *teeth*) suggests that people can rapidly acquire the meaning of words from contexts. In the same vein, we expect that short descriptions of person names will enable people to obtain the meaning of previously unknown names.

Another question is how the acquired meaning of person names is retrieved and integrated into context. It has been well established that context can facilitate the lexical retrieval of words and that people immediately integrate all available information into context [11], [12]. ERPs are ideal for examining the time course of information processing due to their high temporal resolution [13]. The measured ERPs reflect brain responses to certain stimuli, and different ERP components are associated with particular cognitive functions.

A well-known ERP component that relates to meaning processing is the N400. The N400 is a negativity that begins around 200 ms and peaks around 400 ms after stimulus onset, with a centro-parietal maximum distribution [14]. The N400 amplitude varies as a function of how easily a word is integrated into or pre-activated by the previous context. Specifically, semantically unpredicted words elicit a larger N400 than semantically predicted words. The N400 difference has been classified as an N400 effect [15]. The N400 effect was found not only to be sensitive to sentence context, but also to discourse context [16], [17].

In addition to the N400, another ERP component, the P600, was also often reported in language studies. The P600 is a positivity that occurs roughly between 500–1200 ms post-stimulus, with a centro-posterior distribution. The P600 effect is typically found in response to syntactic violations [18], [19], but is also elicited by violations of meaning [20], [21], [22], [23]. A number of studies have observed both N400 and P600 effects in response to semantic violations (for a review see [23]). In addition, a series of studies have reported a P600 effect instead of an N400 effect for semantic verb–argument violations (e.g., *‘The egg eats/is eaten for breakfast.’* ; [20]). Moreover, a P600 effect, rather than an N400 effect, was found when the violating information fits the global context (e.g., *‘After a serious airplane crash, the survivors/victims should be buried properly.’*; [21], [22]). Overall, the P600 effect might reflect prolonged analysis of unexpected inputs [20], [23].

Using ERPs, the present study aims to investigate whether the meaning of person names can be effectively established in short linguistic contexts, and if so, how the meaning of person names is integrated into discourse contexts. We manipulated the congruence of person names in discourses. In each discourse, the first sentence served as an introductory sentence, which introduced the names (e.g., *Laoli* and *Laoma*) as well as a general status (e.g., *being a father*) of two target persons. Then the second sentence described contrastive facts or events of the two target persons (e.g., *Laoli having a son* whereas *Laoma having a daughter*). In the third sentence, a critical name (e.g., *Laoli*) either matched or did not match the previously presented information (e.g., *a boy or a girl who was looking for father was brought to Laoli*). Consequently, the critical name was either congruent or incongruent relative to the discourse context. ERPs elicited by the names were compared between the congruent and incongruent conditions. Some possibilities are envisaged. First, if the meaning of person names can be established by short descriptions and the meaning can be immediately retrieved and integrated into the contexts, the violation of person names will elicit an N400 effect (and probably a following P600 effect). Second, if the meaning is established but not quickly retrieved or integrated, a P600 effect instead of an N400 effect will be expected. Third, no ERP effect will be observed in response to the violation if the meaning of person names is not effectively established by the short descriptive contexts.

## Materials and Methods

### Ethics Statement

The study was approved by the Institutional Review Board of the Institute of Psychology, Chinese Academy of Sciences. All participants provided written, informed consent before taking part in our experiment.

### Participants

Sixteen university students (mean age 22 years, 18–27 years old; 10 males) served as paid volunteers. They were all right-handed native speakers of Mandarin Chinese with normal or corrected to normal vision. None of them had dyslexia or any neurological disorder. No participants were excluded from the study.

### Stimuli

Experimental stimuli comprised three-sentence discourses (See Table 1 for examples). In each discourse, the first sentence served as an introductory sentence, which introduced the names as well as the general status of two targets persons (e.g., *Laoli and Laoma being fathers*). The names were constructed in a way that Chinese words ‘*Lao*’ (meaning ‘old’) or ‘*Xiao*’ (meaning ‘little’) were placed before family names. This way of calling others is very common between friends, colleagues or other acquaintances in China. In this study, ‘Lao’ and ‘Xiao’ were combined with the same set of family names (80 family names for the experimental materials) and referred to 160 different persons. The second sentence described contrastive facts or events of the two target persons (e.g., *Laoli having a son* while *Laoma having a daughter*). The third sentence contained critical information that either matched (e.g. *a boy looking for father was brought to Laoli*) or did not match (e.g. *a girl looking for father was brought to Laoli*) the fact or event of one of the names (e.g., *Laoli having a son*). Therefore, two conditions were created with respect to the names: congruent or incongruent. In addition, the critical name (e.g., *Laoli*) in the third sentence was introduced either in the first or in the second part of the second sentence, so the critical name was either close or far away from its position in the second sentence (hereafter referred to as *distance*, see examples 1 and 3 for the “far” condition, and examples 2 and 4 for the “close” condition in Table 1). Moreover, the critical information that appeared in the target sentence was counterbalanced across conditions for each discourse (e.g., *a boy or a girl looking for father*).

**Table 1:** Examples of four sets of discourses.

1. Congruent/Incongruent
老李和老马都是父亲.老李有个儿子,老马有个女儿.同事把一个找爸爸的小男孩/小女孩带到了老李的办公室.
(Laoli and Laoma are both fathers. Laoli has a son, whereas Laoma has a daughter. The colleague brought ***a little boy/a little girl who was looking for his father*** to Laoli’s office.)
2. Congruent/Incongruent
老卫和老伍都是大学老师.老卫教音乐老伍教美术.一名同学带着自己的绘画作品/作曲来找老伍请教.
(Laowei and Laowu are both colleague teachers. Laowei teaches music, whereas Laowu teaches painting. A student brought ***his painting/his composed song*** to Laowu for advice.)
3. Congruent/Incongruent
小耿和小戴都是初中生.小耿喜欢踢足球;小戴喜欢玩电脑游戏.大家经常在球场/网吧看到小耿的身影.
Xiaogeng and Xiaodai are both junior school students. Xiaogeng likes playing football, whereas Xiaodai likes playing video games. ***In the playground/the internet bar*** people saw Xiaogeng very often.
4. Congruent/Incongruent
小金和小常都很有名.小金是一名歌手,小常是一名电影演员.昨天有一位影视制片人/音乐制片人来找小常商谈合作.
(Xiaojin and Xiaochang are both very famous. Xiaojin is a singer, whereas Xiaochang is an actor. Yesterday ***a film producer/a music producer*** came to Xiaochang for collaboration.)

Note: The examples were originally in Chinese. The English translations are given in brackets below the original Chinese materials. The critical phrases that created violations are in boldface and italicized. The critical names are underlined.

Overall, we constructed 80 sets of experimental discourses, with different discourses containing different characters and describing different scenarios. In each set of discourse, each critical name was presented in eight conditions (i.e., the combinations of congruence, distance, and the critical information that appeared in the target sentence). Note, however, we focused on the Congruence manipulation while disregarding the other two factors in our data analysis due to the limited number of trials in each condition. The eight conditions were distributed across eight experimental lists through a Latin square procedure, with each list containing an equal number of discourses per condition (10 discourses). In this way, all the discourses were presented across the eight experimental lists, and no single participant read the same name more than once. In addition, we exchanged the correspondence between a specific name and its fact or event in order to fully match the physical features of critical names across lists, leading to another eight experimental lists (e.g., *Laoli having a son or a daughter* whereas *Laoma having a daughter or a son*). Overall, there were 16 experimental lists, with 80 discourses per list. Since we focused on the effects of congruence, 40 trials of the same condition resulted per participant.

In order to avoid that participants would only pay attention to one of the names in the context, we created 10 incongruent discourses as fillers where a previously unmentioned name appeared in the target sentence. In this way, participants have to remember both names in the context in order to make correct responses (to judge the congruence of discourses). Additionally, we added 10 congruent discourses as fillers in order to balance the number of congruent and incongruent discourses in one list. These 20 fillers were added to each of the lists.

Taken together, we created 16 lists. Each list consisted of 100 discourses, with 80 experimental discourses (40 congruent and 40 incongruent discourses) and 20 filler discourses.

### Procedure

Participants were seated in a comfortable chair in front of a computer screen. The discourses were presented in white font on a black background, with a font size of 18pt. At the beginning of each trial, a fixation cross was presented in the center of the screen for 1000 ms, followed by a 200 ms blank screen. Then the first two sentences of each discourse were presented one after the other, with an interval of 200 ms in between. The maximum duration of the presentation of the whole sentences was 8000 ms, and the participants could proceed by pressing the space key whenever they wanted. The trial proceeded automatically if the key was not pressed within 8000 ms. After a 200 ms blank screen, the third sentence containing the critical name was presented word by word in order to identify the onset time of the critical name. Each word appeared for 300 ms, with an inter-stimulus interval of 200 ms. Two hundred milliseconds after the presentation of the last word, an instruction was presented in red font on the black background. The participants were instructed to judge the congruence of the whole discourse. They were asked to press one of two keys on the keyboard within 5000 ms: ‘F’ and ‘J’ to be pressed by the left index and right index finger signaling congruent and incongruent, respectively. The next trial began 500 ms after the response. During the experiment, the participants were told to move and blink as little as possible to limit artifacts in the EEG.

The stimuli were divided into 5 blocks in total (20 trials/block), with each block lasting about six minutes. The discourses were presented in a pseudo-random order within each list, with no more than three discourses of the same condition being presented in succession. In between blocks there was a small break, after which the participants could start the next block by pressing a button. The whole experiment lasted about 1.5 hours, including participant preparation, instructions and a short practice consisting of 4 discourses.

### Electroencephalogram (EEG) recording and analysis

The data were recorded by a NeuroScan system, with a cap of 64 electrodes mounted according to the International 10–20 system. The left mastoid electrode served as the reference, and an electrode placed between Fz and Cz electrodes served as the ground. The vertical (VEOG) and horizontal (HEOG) eye movements were monitored through four electrodes placed around the orbital region (bipolar montage). All electrode impedances were kept below 5 KΩ during the experiment. Recording was done with a band pass filter of 0.05 – 100 Hz and a sampling rate of 500 Hz.

The EEG data were re-referenced off-line to the average of both mastoids since this has been used for most N400-related studies. The VEOG artifacts were automatically corrected by NeuroScan software [24]. Data were filtered off-line with a 0.01 – 30Hz (24dB/oct slope) band-pass filter. Given that the retrieval of person names was shown to be completed before 1000 ms [8], critical epochs ranged from 100 ms before to 1000 ms after the onset of the critical names, with 100 ms before the onset serving as the baseline. An automatic artifact rejection procedure was employed to exclude trials for which the amplitude at any one electrode exceeded ±80 µV. Only trials with correct responses were taken into calculation. On average, 36 and 37 trials were kept (the Mean ± SD of the percentage of accepted trials were 90.50%±5.26% and 92.50%±5.74%) respectively for the congruent and incongruent conditions, with no significant difference between the two conditions (F_(1,15)_  =  2.634, p  =  .125, η^2^  =  .149). In the end, the ERPs were calculated by averaging over trials in each condition for each electrode and each participant.

### Statistical analysis

The statistical difference between two conditions was evaluated by a cluster-based random permutation test [25], which was implemented in the Matlab toolbox Fieldtrip [26]. This approach controls the Type-1 error rate that involves multiple comparisons (one comparison for each electrode and each time point). First, for every data sample (electrode*time point) a simple dependent-samples *t* test is performed. All adjacent data samples (spatial or temporal) exceeding a preset significance level (5% here) are grouped into clusters. For each cluster the sum of the *t* statistics is used in the cluster-level test statistic. Then a null distribution that assumes no difference between conditions is created. This distribution is obtained by 1000 times randomly assigning the conditions in participants and calculating the largest cluster-level statistic for each randomization. Finally, the actually observed cluster-level test statistics are compared against the null distribution, and clusters falling in the highest or lowest 2.5^th^ percentile are considered significant. All the time points in the time window of 0 – 1000 ms of all 62 electrodes were entered into the analysis.

## Results

### Behavioral results

We calculated the accuracy as well as the RT of each condition for each participant. Then, the accuracy and RT data were subjected to one-way repeated measures ANOVA, with Congruence serving as a within-subject factor. We found that participants made highly accurate responses in both conditions (Mean ± SD  =  92.5% ± 4.7%; 93.9% ± 5.3% respectively for the congruent and incongruent conditions). The statistical analysis showed no accuracy difference between conditions (F_(1,15)_  =  1.358, p  =  .262, η^2^  =  .083). As for the RT data, participants made faster responses for the congruent condition than for the incongruent condition (F_(1,15)_  =  9.296, p  =  .008, η^2^  =  .383). The mean ± SD of the RT was 767 ms ± 280 and 889 ms ± 257, respectively for the congruent and incongruent conditions.

### ERP results

**Figure 1:**
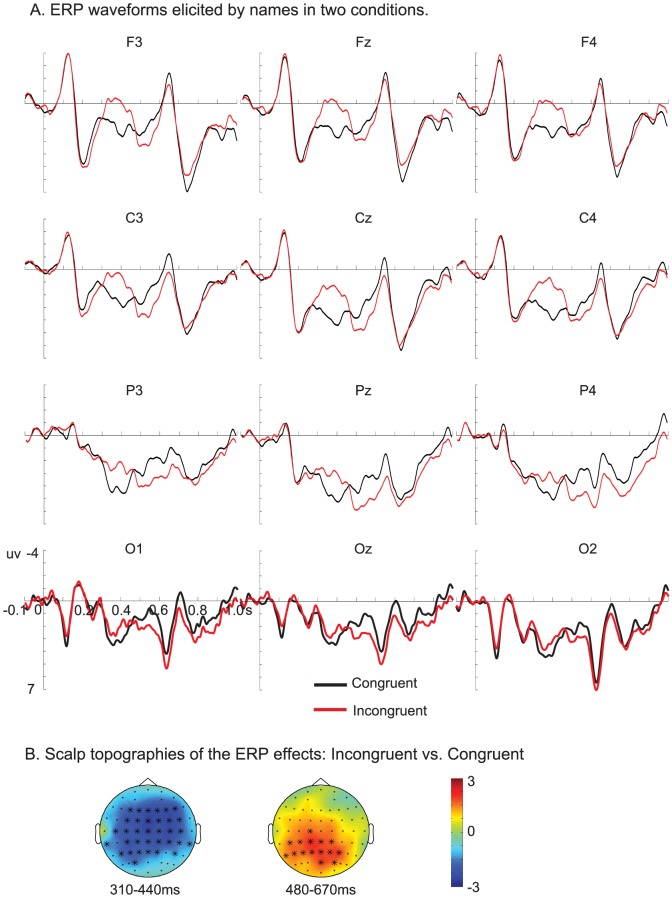
ERP responses evoked by the names. A. Grand averaged waveforms of the two conditions at twelve representative electrodes. Waveforms are time-locked to the onset of the names. Negative is plotted upward. B. Topographies of the congruity effect (Incongruent vs. Congruent) of the critical names in two time windows. The electrodes that showed significant effects over 75% of the selected time windows were marked by *.

Figure 1A displays the grand average ERP waveforms evoked by the critical names in two conditions.

Two significant clusters were revealed in the statistical analysis. One cluster showed a significantly larger negativity for the incongruent than for the congruent condition in the time window of 310 – 400 ms, with a broad distribution (p  =  .011; Figure 1B). The other cluster showed a significantly larger positivity for the incongruent than for the congruent condition in the time window of 480 – 670 ms over posterior regions (p  =  .005; Figure 1B).

## Discussion

This study aimed to examine the integration of newly established meaning of person names into discourse context. Unknown names were associated with different linguistic descriptions. Then the congruence of person names was manipulated in such a way that the meaning of person names was either congruent or incongruent relative to the discourse context. We found that the incongruent names elicited a larger negativity in the time interval of 310 – 400 ms as well as a larger positivity in the time interval of 480 – 670 ms compared to the congruent names.

### Incongruent names elicited a larger negativity than congruent names

Compared to the congruent names, the incongruent names elicited a larger negativity in the time window of 310 – 400 ms. Given its morphology, latency as well as the eliciting condition, we took this negative effect as an N400 effect. The N400 effect has often been reported for common nouns, verbs and adjectives (for a review see [27]). Also, the N400 has been related to lexical retrieval of words [28], integration of words into context [29], or a dynamic interaction between memory retrieval and mental unification [30]. Regardless of the account utilized, the current study clearly showed that the meaning of names could be rapidly activated when it did not fit the contexts, as indicated by the N400 effect in response to the incongruence. It should be noted that the scalp distribution of the N400 effect in the present study appeared to be more prominent over the anterior region, which differs from the classical centro-parietal distribution. A more anterior N400 effect has been found in response to person names [31], gestures [32] and actions [33]. In this regard, the anterior N400 effect found in the current study might be related to the complexity of the stimuli rather than the method of delivery (such as visual, auditory or images). Another possibility is that the N400 effect might partly overlap with the following, posteriorly distributed P600 effect, which resulted in a reduced N400 effect over the posterior region.

The presence of the N400 effect demonstrates that brief descriptions of person names are sufficient to endow meaning to previously unknown names. This finding is in line with contextual word learning studies. For instance, Mestres-Missé et al. (2007) embedded novel words in linguistic contexts from which people could discover the meaning of the novel words. After three exposures of the novel words in different contexts, the ERPs elicited by the novel words were indistinguishable from those of real words [9]. Furthermore, Borovsky et al. (2010) presented novel words in highly constraining contexts only once, and then assessed the meaning of the novel words in a new sentence in which the novel words either fit or violated the meaning of the verbs. They observed an N400 effect when the verbs did not match the meaning of the novel words [10]. In these learning studies, a known concept was mapped onto a novel word form. In the current study, some factual knowledge converged to form the meaning of an unknown name. Regardless, the results indicate that people have an amazing capacity to acquire knowledge from surrounding inputs.

In comparison with previous N400 studies (for a review, see [27]), the comparable effect latency seems to imply that the present N400 effect was not qualitatively different from previous N400 effects elicited by other word categories. This result appears to be inconsistent with earlier findings on single familiar name processing, as it has been shown that the retrieval of a names’ meaning occurs relatively later than that of common nouns [5], [8], [34]. The inconsistency can be accounted for by the context in which the person names were processed. Because name recognition and person identification must be completed before the meaning of a name is retrieved, the retrieval of previously known person names from long-term memory is very demanding in the absence of any context. In contrast, in the current study, the participants obtained the meaning of names from the discourse context, and the meaning of names was still available in working memory by the time of processing the target sentence. Therefore, the incongruence of the discourse can be immediately detected and thus induced an N400 effect. It might be interesting to see whether contexts can also facilitate the integration of previously known names in future studies.

### Incongruent names elicited a larger positivity than congruent names

In addition to the larger negativity, the incongruent names also elicited a larger positivity than the congruent names in the time interval of 480 – 670 ms over posterior regions. Based on its morphology, latency and distribution, we identified this positive effect as a P600 effect. The P600 effect has traditionally been associated with syntactic analysis [18], [19], but this syntactic account cannot be reconciled with the present data because no syntactic information was carried by the names. Previous studies have also reported P600 effects in response to semantic violations [20], [21], [22], [23]. Although the eliciting conditions differed across different studies, one thing in common is that these studies involved prolonged analysis of the stimuli after the N400 time window. The P600 effect has been proposed to reflect disconfirmed predictions [23] or reprocessing of unexpected input under general cognitive control processes [35]. Therefore, the observed P600 effect in the present study might indicate that people actively predicted the incoming names and that they tended to reanalyze the meaning of names when unexpected names were presented. The onset latency of the observed P600 effect (i.e., 480 ms) seems to be earlier than that of previously reported P600 effects (e.g., 500 ms [20], [23], 700 ms [21], and 800 ms [22]). This might be partly due to the use of a cluster-based random permutation test to assess the ERP differences. Unlike previous studies that used fixed time windows of 100 or 200 ms in pre-defined groups of electrodes, the cluster analysis can detect the ERP effects with a better temporal resolution (depending on the data sample rate, 2 ms in the current study) for a relatively smaller number of electrodes that showed the effects. The relatively early onset latency observed in the current study indicates that people were immediately engaged in the reanalysis upon detecting the incongruity of the names.

The P600 effect has been shown to be task dependent, with a larger P600 effect during a sentence plausibility task than in reading for comprehension task [20], [36]. In the current study, we required the participants to judge the congruence of discourses in order to make sure that they were actively engaged in the discourse comprehension process. This task might have engaged the participants for more extensive semantic analysis of the names, and thus the violating names evoked a P600 effect in addition to the N400 effect. In addition, we employed discourses with very similar structures, so the names were highly predictable in the current study. Also, since half of the discourses contained incongruent names, the processing involved in the current study might differ from natural discourse processing. In order to test the generalizability of the results, future studies may consider using more varied and thus less predictable discourses, as well as including less incongruent discourses.

## Conclusions

This study examined the processing of person names in discourse context. We first associated person names with different linguistic descriptions and then measured ERPs elicited by the person names that either matched or mismatched with the previous descriptions. We found that the incongruent names elicited a larger N400 as well as a larger P600 compared to the congruent names, indicating the difficult retrieval or effortful integration of the incongruent names into the previous context. The observed ERP effects indicate that people can rapidly obtain the meaning of unknown names from brief descriptions of the names. Moreover, the immediate detection of the incongruence of person names demonstrates the modulation of context on the processing of person names.
